# Report of the Indiana Hospital for the Insane for the Year 1851

**Published:** 1852-03

**Authors:** 


### ARTICLE VI.

*Report of the Indiana Hospital for the Insane, for the year* 1851. (By favor of Dr. Nutt.)

| | | |
|---|---|---|
| There were in the Institution at the commencement of the year, | 80 | patients. |
| Admitted during the year, | 128 | " |
| Discharged " " " | 71 | " |
| Recovered " " " | 52 | " |
| Died " " " | 13 | " |
| In the Institution at date of Report, | 137 | " |
| Per cent. of recoveries in recent cases (those of less than one year standing) | 80.77 | |
| Per cent. of recoveries in chronic cases, (those over one year standing when admitted, | 55.55 | |

This shows a very favorable result, and must be very gratifying to the friends of the Institution, and the humane and good throughout the State, and speaks well for Dr. Patterson, the medical superintendant under whose direction the treatment of patients is exclusively placed.

By the report of the superintendent we learn that there are, as ascertained by the census of 1850, 442 insane and 617 idiotic persons in the State; and the reasonable supposition is made that many insane have been enumerated as idiotic.

This showing would seem to indicate a necessity for more extensive provisions for the care of the insane, than has yet been made. And the county poor-house system of taking care of the incurable insane is so expensive or else so defective that we think it very properly condemned by the Superintendent.

We have no doubt of the propriety of the State providing Hospital care for all of her insane, yet believe, with a majority of those conversant with the subject, that no one Institution should be extended beyond provisions for 250 patients, which was the original design of this establishment.

The Superintendent thinks it will be capable of accommodating about 300, but when it is recollected that the basement story was only designed to be temporarily occupied for patients, and by but few even temporarily, it will bring the estimates to more closely correspond.

The remarks of the Superintendent upon the medical treatment in the report before us, are judicious, and show a familiarity with the subject, such as we would expect from one of his experience and ability.

We congratulate the people of Indiana upon the success of the Institution, and upon having a free home for the insane, where they may receive the kind attention and skilful management of those who are devoting their whole time and talents to the restoration of the curable, and the amelioration of the condition of those who are unfortunately beyond the hope of recovery.

We can but believe that the steward would find a more extended detail of his disbursements, a safeguard against suspicions of mismanagement, and it would be a satisfaction to the people, who supply freely and liberally the means of supporting the Institution, to know from whom his purchases were made and the prices paid.

